# Life History of *Oxybelus variegatus* Wesmael, 1852 (Hymenoptera: Crabronidae) with a Description of the Mature Larva

**DOI:** 10.3390/insects12020100

**Published:** 2021-01-24

**Authors:** Piotr Olszewski, Petr Bogusch, Krzysztof Szpila

**Affiliations:** 1 Department of Ecology and Biogeography, Faculty of Biological and Veterinary Sciences, Nicolaus Copernicus University, Lwowska 1, 87-100 Toruń, Poland; Krzysztof.Szpila@umk.pl; 2 Department of Biology, Faculty of Science, University of Hradec Králové, Rokitanského 62, 500 03 Hradec Králové, Czech Republic; bogusch.petr@gmail.com

**Keywords:** bionomics, ecology, digger wasp, *Oxybelus variegatus*, larval morphology, kleptoparasites

## Abstract

**Simple Summary:**

Poor knowledge of the morphology of larvae and natural history data on the genus *Oxybelus* prompted us to research the rare digger wasp *Oxybelus variegatus*. We have described the mature larva for the first time and analysed the nesting behaviour of females, nest structure, prey range, phenology and accompanying kleptoparasites. Our findings indicate that this endangered species may inhabit strongly anthropogenic habitats and shows a potentially effective role in controlling dipteran crop pests such as *Delia platura*.

**Abstract:**

The first comprehensive information on the bionomics of the digger wasp *Oxybelus variegatus* Wesmael, 1852 is presented. Females nested in small aggregations in crevices between paving stones of a frequently used pedestrian pathway in lowland agricultural wasteland. Nests were dug in the ground using mandibles, legs and abdomen. The nest consists of a main burrow with one or, rarely, two cells. The mature larva is described for the first time. The egg stage lasts for about two days before the larva hatches. The female provisioned each cell with an average of 11 paralysed male flies of *Delia platura* (Meigen, 1826) (Diptera: Anthomyiidae). Numerous females of dipteran kleptoparasites were observed in the nesting area of *O. variegatus*. However, only a few nests were infested by larvae of *Senotainia conica* (Fallen, 1810).

## 1. Introduction

The genus *Oxybelus* Latreille, 1796 is widespread throughout the world, except Australia, and includes 264 species [1], of which 25 species occur in Europe [2] and 12 in Poland [3]. *Oxybelus* are small wasps (4–11 mm long), stockily built, often with a pattern of yellow or white spots or stripes on the abdomen. Most species prefer dry, sunny and sandy areas. Females dig the main burrow that ends with one or two cells at a depth of about 6–10 cm [4]. They prey on adult flies, mostly Anthomyiidae, Calliphoridae, Muscidae and Sarcophagidae [5]. The nest entrance remains open or is closed during provisioning of the cell, depending on the species. The paralysed prey is transported by the female, either impaled on the sting or held with middle or hind legs [4]. Species of *Oxybelus* also prey on species of economic and medical importance [6]. Information on the biology of *Oxybelus* is available in many publications, but in most cases, the data provided are scarce and general [7,8,9,10].

Mature larvae have been described for four Palaearctic species [11]: *O. haemorrhoidalis* Olivier, 1812 by Grandi [12]; *O. argentatus* Curtis, 1833 by Grandi [13]; *O. lamellatus* Olivier, 1811 and *O. spectabilis* Gerstaecker, 1867 by Asís et al. [11]. Larvae of two Holarctic species were also described: Maréchal [14,15] and Evans [16] described the larva of *O. bipunctatus* Olivier, 1812, while Evans [16] described the larva of *O. uniglumis* (Linnaeus, 1758). The latest publications about the behavioural ecology and biology of the genus are summarised by Andrietti et al. [17], Blösch [18], Packeman et al. [9] and Tormos et al. [19]. Information on the larval stages remains scarce and the present paper is a step towards improving this situation.

Blösch [18] reported that *O. variegatus* occurs and nests only in grey hair-grass (*Corynephorus canescens* L.) and among herbaceous plants on clay-sandy soil along noise barriers in Erlangen, Germany. Nicoli Aldini [20] described the nesting habits of *O. variegatus* in the Camonica Valley (Lombard Alps, northern Italy). The nests consisted of a burrow ending in a single cell. They remained open while females were hunting and mass provisioning with flies of the family Anthomyiidae. Grandi [12] lists three other families of prey: Muscidae, Tachinidae and Sarcophagidae. Bitsch and Leclercq [21] presented a list of plants visited by the imago of *O. variegatus*.

The objective of the present study is to provide, for the first time, a description of the mature larva of *O. variegatus* and to supplement the existing information on the nesting biology, including the (1) nesting behaviour of females, (2) male mating behaviour, (3) structure of the nest, (4) prey range, (5) phenology and (6) accompanying kleptoparasites.

## 2. Materials and Methods

The study was conducted in the town of Kowalewo Pomorskie (53°10’05.7” N; 18°52’15.5” E) in northern Poland, from early June to early August 2020 on sunny and warm days (with a temperature of at least 18 °C).

The study was carried out in a 26-m^2^ backyard paved with cobblestones. It is located near a wasteland (abandoned arable land) overgrown with herbs and featuring several sandy patches. The distance between the backyard and the wasteland was about 6 m. The dominant plant species growing in the surroundings of the study area were *Tanacetum vulgare* L., *Taraxacum officinale* F.H. Wigg., *Geranium pusillum* L., *Trifolium arvense* L., *Lactuca serriola* L., *Cerastium holosteoides* Fr. em. Hyl., *Berteroa incana* (L.) DC., *Artemisia vulgaris* L., *Achillea millefolium* L. and *Potentilla anserina* L.

The behaviour of both sexes, digging of nests and the frequency with which the female provisioned the cells were analysed on the basis of videos recorded with a Canon Camera CCD-V800E 10X and by direct observations. An additional Raynox Macroscopic Lens M-250 was also used to take photographs. The structure of the nest was analysed by removing the paving stones. The prey range was determined by nest inspections. To describe the larval specimens, we transferred some of the larvae into Pampel solution (30 volumes of distilled water, 15 volumes of 96% ethanol, 6 volumes of formaldehyde and 4 volumes of glacial acetic acid) as described by [22], while the remaining larvae were allowed to grow to the adult stage. After taking photographs of the intact larvae, we examined their sclerotised parts. For this purpose, we placed the larvae into 10% solution of hot (60 °C) KOH for 12 h to dilute all body parts except the integument. We then coloured the integument in 5% Chlorazol Black E (Sigma Aldrich) for 2 s and then moved it into 96% ethanol. To observe the species-specific characteristics, we placed the integument into glycerol and separately observed the head, mouthparts, spiracles and other parts under a light microscope. We used the same specimens to study small structures such as setae, sensilla or mouthparts. We drew figures of (1) the head, with a focus on the clypeus, labrum, maxillae and labium; (2) mandibles—anterior view; and (3) spiracles of larvae. Specimens of kleptoparasitic flies and prey are deposited at the Department of Ecology and Biogeography of Nicolaus Copernicus University, Poland, and larvae are deposited at the University of Hradec Králové, Czech Republic. Larvae and pupae of kleptoparasitic flies and digger wasps were reared in Eppendorf tubes until they developed into imago. Breeding of digger wasps under laboratory conditions from the egg stage to pupae was not successful.

## 3. Results

### 3.1. Environmental Preferences and Nesting Behaviour

**Figure 1:**
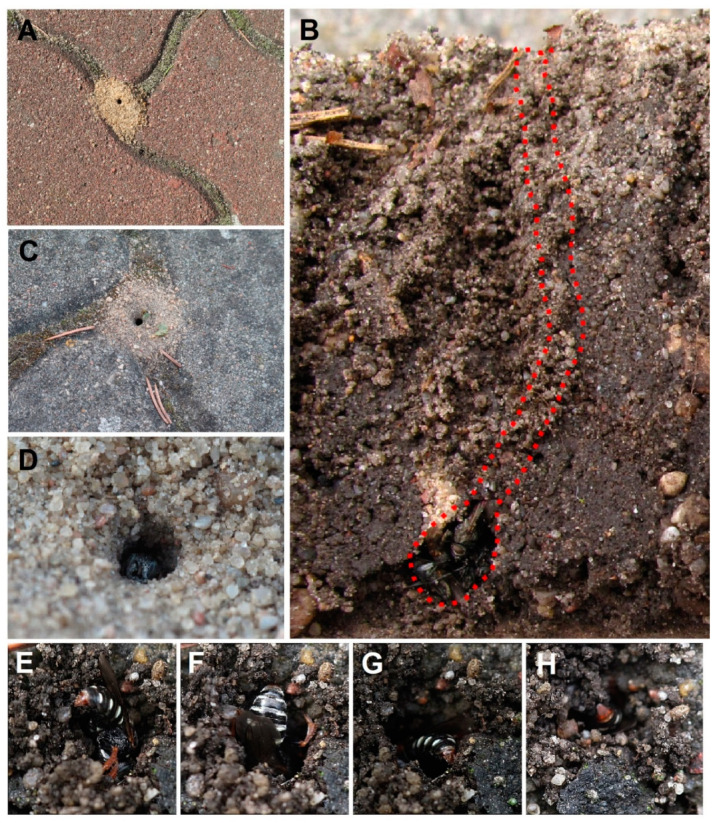
Nest of *Oxybelus variegatus*. (**A**,**C**,**D**) Top view of the nest entrance; (**B**) cross-section of the nest after removing a single paving stone; (**E**–**H**) burrow excavation.

**Figure 2:**
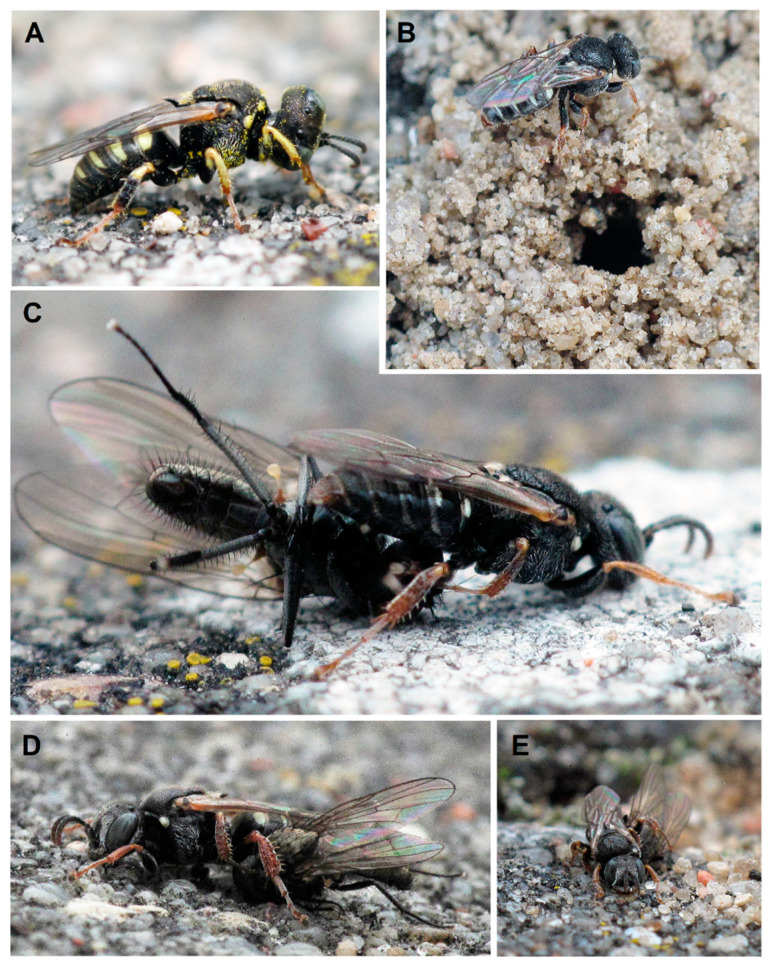
Adult of *Oxybelus variegatus*. (**A**) Male; (**B**) female in front of the nest entrance; (**C**–**E**) female with prey.

Behavioural data. From early June to mid-July, females used gaps between paving stones to dig 32 nests (north-eastern aspect; Figure 1A–H), while from mid-July to early-August, nests were also observed in adjacent areas, in the sandy area (four nests) and in the wasteland (six nests). Eight females provisioning cells with adult Diptera were observed simultaneously at the peak of the nesting period. Females were observed from 10:00 to 20:15 h, while males (Figure 2A) were most frequently flying low above the ground from 10:00 to 15:00 h (2 or 3 cm above the ground) in a twisting flight. During that time, fighting ensued when a male approached another too closely, while females were single, and copulation took place when both sexes came into contact. No copulation was observed after 15:00 h. In total, we observed three copulations lasting approximately 2 min on the ground.

Females (Figure 2B–E) nested in dense aggregations in summer and selected areas without vegetation to build their nests. Before selecting a nesting site, females briefly landed for test digging while flying close to the ground.

To start a nest, the female first loosened the soil crust with her mandibles and forelegs. Then, her forelegs raked the loosened soil under the raised abdomen. The female repeatedly retreated from the burrow, pushing loads of sand held between the abdominal venter and hind legs (Figure 1E–H). The excavated soil formed a small mound around the nest entrance (Figure 1A,C).

**Figure 3:**
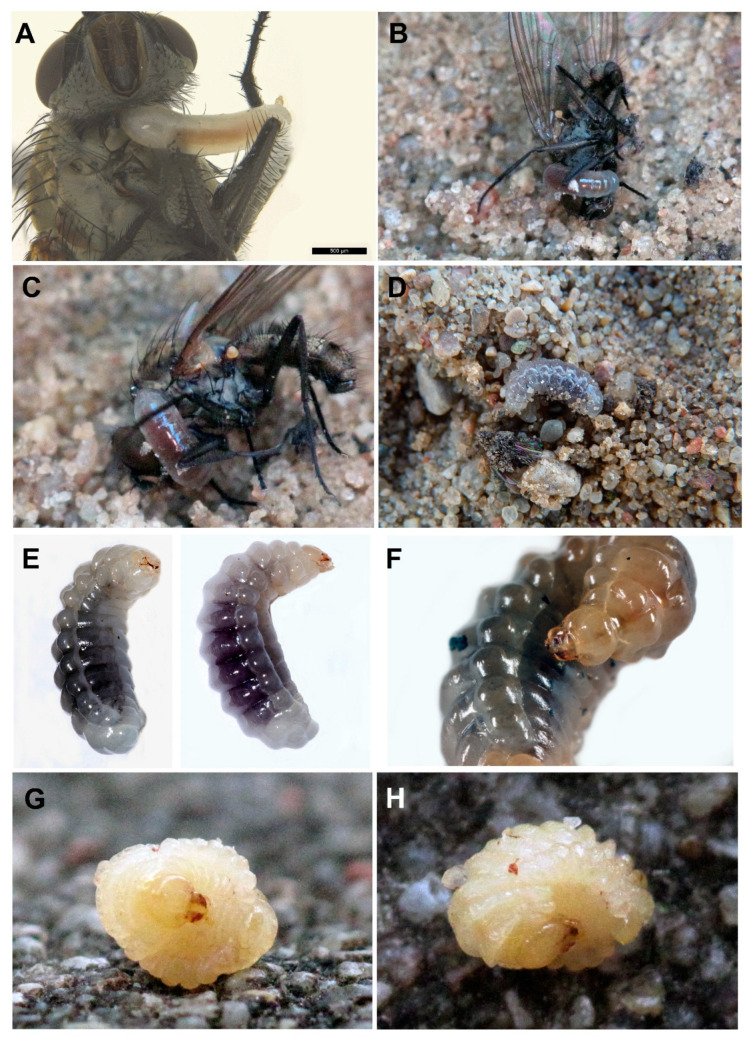
Developmental stages of *Oxybelus variegatus*. (**A**) Egg; (**B**–**H**) larva.

Ten nests of the colony were explored (all in the backyard area with cobblestones). The length of the main burrow was approximately 5–7 cm, with a diameter of about 2.5–3 mm, and its course was basically vertical halfway along its length, becoming oblique afterwards and ending with a single cell (N = 8) or, rarely, two cells (N = 2) with a length of about 12 mm and a width of 5–7 mm (Figure 1B). On average, 11 paralysed male flies of *Delia platura* (Meigen, 1826) were stored in each cell (Figure 1B; Figure 3B–C). The field with beans, on which *Delia platura* flies probably develop, was located about 6 m from all breeding sites. The minimum distance between nest entrances was about 7 cm. The entrance to the burrow was constantly open during the provisioning period. Females were observed repeatedly with their heads facing outside the entrance (Figure 1D). Females always transported their prey from the left side, impaled on their sting on the side of the thorax (Figure 2C–E), sometimes, after landing near the entrance, holding the prey close to the side using the middle legs. The provisioning female landed in front of the entrance and entered immediately. The time interval between bringing two successive prey was 2–24 min (usually 4–6 min).

The development of *O. variegatus* is presented in Figure 3A–H. A white oblong egg was laid on the ventral side of the fly and the egg placement can be classified as Crabro-style after Iwata [23] (Figure 3A). It was 1.567 mm long and 0.359 mm wide (N = 10). In 70% of the cases, the egg was laid on the right side of the prey. The egg stage took about two days. Breeding of the larva from the egg stage to pupa was not successful.

**Figure 4:**
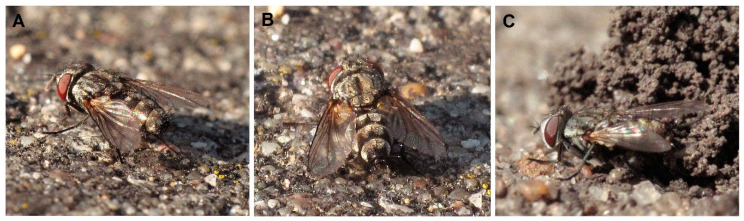
Kleptoparasites. (**A**,**B**) Females of *Metopia argyrocephala*; (**C**) female *Senotainia conica*.

Kleptoparasitic flesh flies of *Metopia argyrocephala* (Meigen, 1824) (N = 6; Figure 4A–B) entered the nests in the absence of the female (all observations conducted in the backyard area with cobblestones) and *Senotainia conica* (Fallen, 1810) (N = 7; Figure 4C) approached the nests shortly after the female wasps returned with prey (all observations conducted on wasteland—N = 2 and sandy patches—N = 5). Out of all analysed nests, two larvae of *S. conica* collected in the nests from an adjacent area (sandy spot) were reared to adulthood and subsequently identified to species. More than one fly larva was not observed in the nest cell.

### 3.2. Description of the Mature Larva

#### 3.2.1. Material Examined

**Figure 5:**
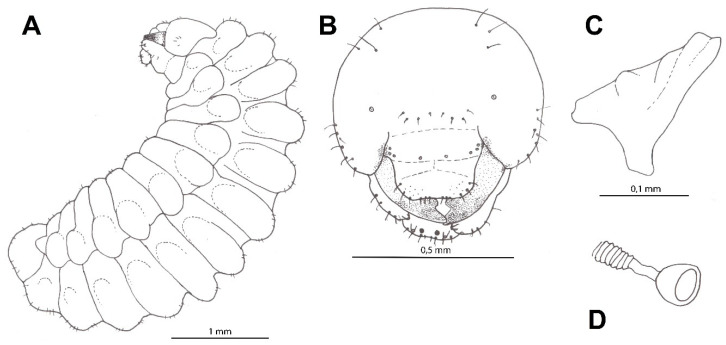
Larva of *Oxybelus variegatus*: (**A**) body, lateral view; (**B**) head, frontal view; (**C**) mandible, frontal view; (**D**) spiracle.

Two specimens (Figure 5A–D).

#### 3.2.2. Diagnosis

The mature larva of *O. variegatus* is similar to other larvae of this genus, with a fusiform body, slightly thicker body posteriorly (Figure 3E–F), conspicuous lateral tubercles and large, elongated mandibles with five teeth. It is small and resembles the larva of *O. bipunctatus* in size, from which it differs in having blunt apical teeth on the mandibles (like *O. argentatus*). The mature larva has many setae around the mandibular condyle. The mandible is brown and sclerotised as well as maxillary and labial palpi; the rest of the mouthparts are not pigmented.

#### 3.2.3. Description

Body length: 5.8–7.1 mm (N = 2). Body colour: light yellow (prepupae) or cream (larvae; Figure 3B–H). Integument with sparsely distributed short spicules and several slender, pale setae, tapering to fine points, arising from small but distinct alveoli; setae not elongated. Several setae on mouthparts; only mandible, the area around mandibular condylus as well as maxillary and labial palpi brownish. Other body parts with only a few setae, except last three metasomal segments—more setose. The body form of post-defecating larva, fusiform, robust and only slightly dorsoventrally flattened; body segments of similar width along whole length (Figure 5A). Paired body tubercles present and well developed on all mesosomal and metasomal segments except on T10; T3–T6 with most conspicuous tubercles compared to other terga, while very small on T9. Paired body tubercles on prothorax flattened and elongated, different from those on other body segments. Dorsal tubercles wide, flat and well developed on all three thoracic segments and abdominal terga, while less conspicuous on T9. Predefecating larva in side-view with first abdominal segments largest in diameter and its outline tapering slightly forwards and backwards from there. Abdominal segment 9 more hirsute than previous one, and segment 10 attached to middle of segment 9 in lateral view; anus positioned medially and transversely. Spiracles (Figure 5D) unpigmented, subequal in diameter; atrium globular, slightly wider than deep, projecting little above body wall, with rim; atrial opening diameter 0.75× peritreme width; atrial inner surface with rows of wrinkles concentric with primary tracheal opening; primary tracheal opening without collar; subatrium short, with about 10 chambers of approximately equal size except one or two next to atrium slightly larger in diameter. Sex characters unknown.

Head: Head is moderately small compared to whole body; oriented in normal, hypognathous position relative to the thorax. Setae are long but sparse on the upper part of head capsule; those of maxillary and labial apices are large, straight and conspicuous. Head capsule is unpigmented except at articulation with mandible; mandible is moderately pigmented except mandibular apices and articulation with head capsule conspicuously pigmented; maxillary sclerites faintly pigmented; salivary lips projecting but unpigmented; maxillary and labial palpi all uniformly moderately pigmented (Figure 5B). Coronal and postoccipital ridges absent. Tentorium mostly absent because of impending ecdysis. Parietal bands are absent. In lateral view, clypeus is roundly projecting beyond frons, antenna arising from ill-developed prominence and labrum extending beyond clypeus. Diameter of basal ring of antenna is less than 1/5 of distance from the nearest point on ring to the centre of anterior tentorial pit; antennal papilla only slightly pigmented, very small and not elongated, bearing two sensilla apically. Frontal area between antennae with two linear rows of four setae. Parietal region with three setae from pleurostomal ridge to anterior tentorial pit and multiple on sides. Clypeus is wide with ill-developed basal and apical margins; two sensilla basally onside and three small sensilla more medially on each side. Labrum shallowly emarginated apicomesally, with a group of six conspicuous setae and several smaller sensilla on each side apically; labral sclerite is ill-defined and only very slightly pigmented. Epipharynx is simple with small spinulae laterally. Mandible is moderately robust, darkly pigmented, pentadentate with two blunt apical teeth and three tubercles; medial tooth with fairly sharp apex; proximal tooth blunt and well developed; outer mandibular surface is without setae (Figure 5C). Maxillary apex strongly bent mesad in frontal view, so that maxillary palpus looks subapical; cardo distinct, posterior end directed towards posterior tentorial pit; stipes sclerotised; maxillary palpus elongated, more than twice as long as its basal diameter, both pigmented. Stipes with four conspicuous setae. Labium not divided into prementum and postmentum; apex moderately narrow in frontal view. Two setae on each side and two smaller on ventral surface of labium. Salivary lips round and well visible, with inner surface bearing parallel longitudinal grooves; width of lips slightly more than twice the width of maxillary palpus. Labial palpus elongated with three sensilla in at mid-length.

## 4. Discussion

The biology of European *Oxybelus* Latreille, 1796 is incompletely known and the available information is mostly limited to dipteran prey for larvae, to plants as a source of nectar for adults and to environmental preferences [18,21,24,25,26].

Our data on *O. variegatus* on the nest structure, prey for larvae and breeding behaviour of females mostly agree with observations by Nicoli Aldini [20].

Nicoli Aldini [20] reported that the maximum number of prey in one cell was eight specimens (Anthomyiidae). In our study, this number was not less than 11 individuals per cell. The difference may depend on the species and size of captured flies.

The observations presented in this paper indicate that *O. variegatus* tolerates various types of subsoils for the construction of nests. The choice of an unusual place in crevices of paving stones to build a nest may be associated with appropriate thermal conditions (shaded area from 17:00 h) and a smaller number of kleptoparasites in this type of microhabitat. From mid-July to early August, nests were also built in the neighbouring wasteland, where at least several dozen parasitic flies were observed. Seven of them were caught and identified as *S. conica*, and two larvae of this species were subsequently found in the nests of *O. variegatus.* The low level of nest infestation by kleptoparasites is surprising in the context of the lack of temporary closure of the nest entrance by *O. variegatus*. This phenomenon may be related to the observed nest-guarding behaviour described also for Nearctic *Oxybelus subulatus* Ch. Robertson, 1889 [27]. Counter-kleptoparasitic behaviour was used by *O. subulatus* against flies classified as “hole searchers” and “satellites” [28], which corresponds well to the kleptoparasitic strategies of Miltogramminae species persecuting *O. variegatus*. In the case of *O. variegatus*, however, this nest-guarding behaviour was performed only by females and not by males, as is known for *O. subulatus*.

The upper burrow profile is vertical and similar to the nest structure of Nearctic *Oxybelus* [29]. This group includes *O. sparideus, O. sericeus, O. subulatus, O. cressonii, O. emarginatus* and *O. subcornutus.* Other species with described nests build the upper at an oblique angle. The nesting behaviour of *O. variegatus* is similar to that of Nearctic species [9] in the following aspects: (1) lack of temporary closure, (2) females impale the prey on their sting during transport and entry into the nest and (3) females remove sand using their abdomen during the next excavation.

Females of *Oxybelus* capture flies that are available and abundant near the nesting area. Some species catch only males of certain fly families [5,17,20]. This is consistent with our observation, where only males of *Delia platura* (Anthomyiidae) were the prey. The field with beans, on which *Delia platura* flies could develop, was located about 6 m from all breeding sites. It is likely that catching only male specimens was associated with their high concentration at the same time, e.g., during the swarm behaviour [30]. It is evident that the species shows some variation in the choice of prey. Gayubo and Tormos [31] list *Musca domestica* Linnaeus, 1758 as food for larvae, while Grandi [12], in addition to *Stomoxys calcitrans* (Linnaeus 1758), lists *Phania curvicauda* (Fallen 1820), *Miltogramma brevipilum* Villeneuve, 1911 and *Phrosinella nasuta* (Meigen, 1824), known as parasitoids or the nest parasites of other insects.

The number of European *Oxybelus* species with described larvae is limited [11]. However, the available data indicate morphological homogeneity of all known larvae [11,12,13,14,15,16]. This is well expressed, especially in the form of epipharynx, whose apicomedian area is smooth and without spinules [11]. The spinules are positioned mostly on the base sides, and the lacinial area is both papillose and spinulose. This characteristic is present in all known larvae of *Oxybelus* [11]. Within the subfamily Crabroninae, differentiates the larvae of Crabronini and Oxybelini [16]. However, this area is much smaller in *O. haemorrhoidalis* [11,12]. Asís et al. [11] indicate another characteristic common to all species—setae running across the ventral side of the labrum, which is also developed in *O. variegatus*.

*Oxybelus variegatus* is included on the red lists of endangered invertebrates in Poland and Germany with the status Endangered and Vulnerable, respectively [32,33]. Therefore, it is quite surprising to observe successful nesting of this species in such an anthropogenic habitat such as frequently used paving stone paths. Our observation also indicates the importance of small sandy areas in the agricultural landscape. They are used by digger wasps as nesting sites, which may have a beneficial effect on biological control of pest species [34,35].

## 5. Conclusions

Females of *O. variegatus* usually dig their nests between 12:00 and 15:00 and make provisions between 15:00 and 20:15. The only prey were males of *Delia platura.* They transport the prey on the left side, impaled on the sting. The main burrow is almost vertical along upper half of its length, then oblique, ending with one or, rarely, two cells. The entrance to the main burrow is permanently open during provisioning. The egg is laid on the ventral side of flies and the egg placement can be classified as Crabro-style. The larva of this species is smaller in size compared to the known larvae of most species of this genus. The body shape is typical for *Oxybelus* larvae—fusiform, with well-developed lateral and dorsal tubercles, small spinulae on the cuticle and a relatively small head with elongated mouthparts and mandibles with three apical and two lateral teeth. Unlike most larvae of *Oxybelus*, the mature larva of *O. variegatus* has blunt and short apical teeth (similar to the larva of *O. argentatus*). Our research indicates that this species adapts to anthropopressure and plays a possible role in controlling dipteran crop pests such as *Delia platura.*

## Data Availability

The data that support the findings of this study are available from the corresponding authors, upon reasonable request.
